# Supplementary material for: Technological evolution of large-scale blue hydrogen production toward the U.S. Hydrogen Energy Earthshot
Source: Nat Commun. 2024 Jul 6;15:5684. doi: 10.1038/s41467-024-50090-w (PMC11227542; doi:10.1038/s41467-024-50090-w)
Supplement: Supplementary file 1 — Supplementary Information [file 41467_2024_50090_MOESM1_ESM.pdf]

Supplementary Information

Technological evolution of large-scale blue hydrogen production toward the U.S. Hydrogen Energy Earthshot

Wanying Wu[1], Haibo Zhai[1,2,3,*] & Eugene Holubnyak[2]
[1]College of Engineering and Physical Sciences, University of Wyoming, Laramie, WY 82071, USA
[2]School of Energy Resources, University of Wyoming, Laramie, WY 82071, USA
[3]Department of Engineering and Public Policy, Carnegie Mellon University, Pittsburgh, PA 15213, USA

*Corresponding Author Email: hzhai@uwyo.edu; Phone: 307-766-2318

Number of Supplementary Tables: 18
Number of Supplementary Figures: 6
Number of Pages: 24

This supplementary information consists of eight notes: (1) fossil fuel-based hydrogen production plant characteristics, (2) cost of CO_2 avoided by blue and green hydrogen, (3) subsystems cost allocation, (4) initial unit cost and installed capacity calculations, (5) fossil fuel-based hydrogen production life cycle emissions and tax credits, (6) natural resources consumption, (7) development of a time-based diffusion model of low-carbon hydrogen, and (8) hydrogen production cost estimation in nominal dollars.

Supplementary Note 1: Fossil Fuel-Based Hydrogen Production Technologies

Supplementary Note 1 summarizes the two hydrogen production plant characteristics (Supplementary Table 1), natural resources consumption intensity (Supplementary Table 2), and configurations (Supplementary Figures 1 and 2). These characteristics, such as capacity factors, hourly hydrogen production flow rate, and book lifetime, are used to calculate the levelized cost of hydrogen.

Supplementary Figures 1 and 2 show the configurations of steam methane reforming with carbon capture and storage (SMR-CCS) and coal gasification with carbon capture and storage (coal-gasification-CCS), respectively, which are derived from the National Energy Technology Laboratory (NETL)'s report on state-of-the-art technologies for blue hydrogen production[1]. In Supplementary Fig. 1, SMR-CCS is decomposed down into five subsystems, while coal-gasification-CCS is decomposed into nine subsystems (Supplementary Fig. 2). The detailed definitions of subsystems for both technologies are also included in Supplementary Tables 3 and 4. The capital and operating and maintenance (O&M) costs of individual subsystems and their initial installed capacity are discussed later.

Supplementary Table 1. Technologies and economic characteristics[1].

Sections	Parameters	Unit	Steam Methane Reforming with Carbon Capture and Storage	Coal Gasification with Carbon Capture and Storage
Base Plant	Feedstock	Unitless	Natural Gas	Illinois No.6 Coal
	Gross Output	MW_e	0	109
	Parasitic Load	MW_e	41	148
	Net Purchased Power	MW_e	41	39
	Capacity Factor	%	90%	80%
	Dollar Year	Unitless	2018	2018
Hydrogen Purification	Hydrogen Purification Configuration	Unitless	Pressure Swing Adsorption	Pressure Swing Adsorption
	H_2 Purity	% V-L Mole fraction[a]	99.98%	99.90%
	H_2 Product Pressure	MPa	6.48	6.48
	H_2 Production Flow Rate	kg/hour	20,125	27,500
		kg/day	483,007	660,004
		kg/year	158,667,800	192,721,168
Carbon Capture System	Process Configuration	Unitless	Methyl Diethanolamine and Cansolv	Dual-stage Selexol
	Overall Carbon Capture Efficiency	%	96.2%	92.5%
	CO_2 Purity	% V-L Mole fraction[a]	99.95%	99.08%

	CO_2 Product Pressure	MPa	15.3	15.3
	CO_2 Production Flow Rate	kg/hour	192,413	456,364
	Total CO_2 Transport and Storage Cost	\$/metric ton CO_2	10	10
Financing and Economic Parameters at a Plant Level	Fixed Charge Rate[b]	fraction/year	0.059	0.059
	Book Lifetime	years	30	30
	Fuel Price	\$/GJ; \$/metric ton	4.2	57.3
	Electricity Price	$\$/MWh_e$	71.7	71.7
	Total As-Spent Capital	\$	896,662,000	4,452,975,000
	Annual O&M cost (Including CO_2 Transportation and Storage Cost)[c]	\$/year	207,261,798	334,351,266
	Levelized Capital Cost	$\$/kg H_2$	0.33	1.35
	Levelized Operating and Maintenance Cost (Including CO_2 Transportation and Storage Cost)	$\$/kg H_2$	1.31	1.73
	Levelized Cost of Hydrogen (Including CO_2 Transportation and Storage Cost)	$\$/kg H_2$	1.64	3.09

[a]V-L stands for vapor liquid portion of stream (excluding solids)[1].
[b]The value is calculated using the method from NETL (2011 and 2021)[2,3], as discussed later.
[c]The value is the summation of fixed operating and maintenance cost, non-fuel variable operating and maintenance cost, fuel cost, and the CO_2 transportation and storage cost. The CO_2 transportation and storage cost is calculated in terms of hourly captured CO_2 flow rate, transport and storage cost, plant's capacity factor, total annual hours (8760 hours).

Supplementary Table 2. Natural resources consumption intensity[1].

Items	Unit	Steam Methane Reforming with CCS	Coal Gasification with CCS
Fuel Feedstock	$kg/kg H_2$	3.75	7.71
Total Water Withdrawal	$m^3/kg H_2$	3.07E-02	3.01E-02
Total Water Consumption	$m^3/kg H_2$	2.41E-02	2.36E-02
Total Gross Output (Electricity)	$kWh_e/kg H_2$	0.00	3.96
Total Parasitic Load (Electricity)	$kWh_e/kg H_2$	2.04	5.38
Total Net Power Purchased (Electricity)	$kWh_e/kg H_2$	2.04	1.42
Total Captured CO_2	$kg CO_2/kg H_2$	9.56	16.6
Total CO_2 Direct Emission	$kg CO_2/kg H_2$	0.38	1.35
Land Use	$m^2/kg H_2$ per year	2.55E-03	6.30E-03

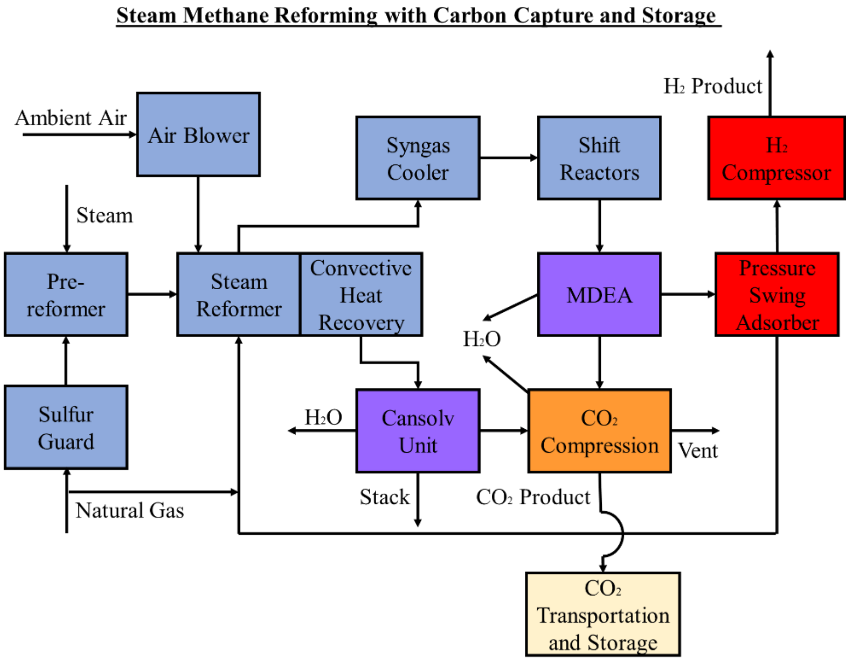

Supplementary Fig. 1. Process diagram of steam methane reforming with carbon capture and storage[1]. Note to abbreviation: MDEA means methyl diethanolamine.

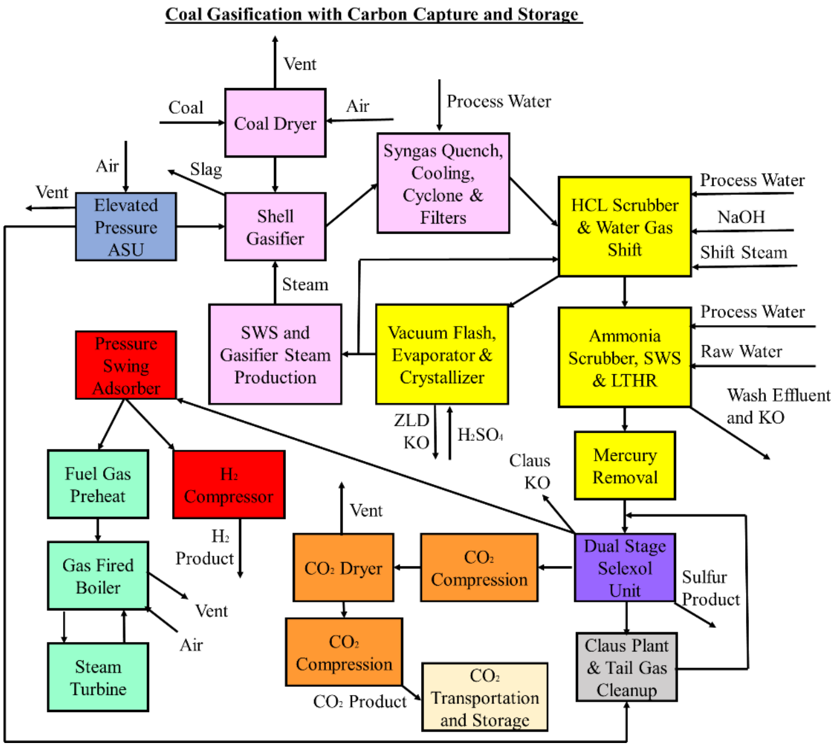

Supplementary Fig. 2. Process diagram of coal gasification with carbon capture and storage[1]. Note to abbreviations: ASU means air separation unit; KO means knockout from an equipment; LTHR means low temperature heat recovery; SWS means sour water stripper; and ZLD means zero liquid discharge.

Supplementary Table 3. Subsystems of steam methane reforming with carbon capture and storage.

Subsystems Defined in This Study	Components Defined in the NETL Study[1]
Pressure Swing Adsorption	Pressure Swing Adsorber, Hydrogen Compressor
Steam Methane Reforming	Sulfur Guard, Pre-reformer, Convective Heat Recovery and Steam Reformer, Syngas Cooler, Shift Reactors, Air Blower
CO_2 Capture	Methyl Diethanolamine (MDEA), Cansolv Unit
CO_2 Compression	CO_2 Compressors, CO_2 Dryer
CO_2 Transportation and Storage	CO_2 Transportation and Storage

Supplementary Table 4. Subsystems of coal gasification with carbon capture and storage.

Subsystems Defined in This Study	Components Defined in the NETL Study[1]
Air Separation Unit	Elevated Pressure ASU
Gasification Block	Coal Dryer, Shell Gasifier, Sour-Water-Stripper and Gasifier Steam Production, Syngas Quench Cooling, Cyclones & Filers
Syngas Cleanup	HCL Scrubber & Water Gas Shift, Ammonia Scrubber, Sour-Water-Stripper & Low Temperature Heat Recovery System, Mercury Removal, Vacuum Flash, Evaporator & Crystallizer, Process Water
Sulfur Recovery	Claus Plant & Tail Gas Cleanup
Pressure Swing Adsorption	Pressure Swing Adsorber, H_2 Compressor
Power Block	Fuel Gas Preheat, Gas-Fired Boiler, Steam Turbine
CO_2 Capture	Dual-Stage Selexol Unit
CO_2 Compression	CO_2 Compressors, CO_2 Dryer
CO_2 Transportation and Storage	CO_2 Transportation and Storage

Supplementary Note 2: Cost of CO_2 Avoided by Blue and Green Hydrogen

The cost of CO_2 avoided by blue or green hydrogen relative to grey hydrogen can be estimated in terms of the production plant's CO_2 emission intensity and levelized cost of hydrogen:

$$\mathrm{CCA} = \frac{\mathrm{LCOH_{blue/green}} - \mathrm{LCOH_{grey}}}{\mathrm{EI_{grey}} - \mathrm{EI_{blue/green}}} \tag{S1}$$

Where CCA is the cost of CO_2 avoided ($/kg CO_2); LCOH is the levelized cost of hydrogen ($/kg H_2); EI is the stack CO_2 emission intensity of a production plant (kg CO_2/kg H_2); the subscript "blue" represents the gas-based blue hydrogen production, the subscript "green" represents the green hydrogen production, and the subscript "grey" represents the gas-based grey hydrogen production without carbon capture.

Supplementary Table 5 summarizes the stack CO_2 emission intensity and LCOH of grey, blue, and green hydrogen production plants, which are collected from the literature[1,4]. The resulting costs of CO_2 avoided by blue and green hydrogen relative to grey hydrogen are also provided in Supplementary Table 5.

Supplementary Table 5. Hydrogen production emission, production cost, and carbon avoidance cost.

Production Technology	Stack Emission Intensity ($kg\ CO_2/kg\ H_2$)	Levelized Cost of Hydrogen (2018$/kg H_2)	Cost of CO_2 Avoided ($/metric ton CO_2)
Steam Methane Reforming (SMR) (Grey)	9.35	1.06	Reference
SMR with CCS (Blue)	0.38	1.64	65
Distributed Polymer Electrolyte Membrane Electrolysis (Green)	0[a]	5.17 (3.04–7.50)[b]	440 (212-689)

[a]Zero carbon emissions are assumed for the electrolysis powered by solar photovoltaics as it produces almost no direct emissions during operation[5].

[b]The cost was estimated for a production capacity of 1,500 kg H_2/day with an effective electricity price of 7.55 ₵/kWh[4]. In the probabilistic analysis, the uncertainties are considered for parameters such as electricity price, stack electrical usage, stack cost, stack replacement interval, electrical balance of plant cost, mechanical balance of plant cost, stack replacement cost percentage, and capacity factor[4]. Electricity price is ranged from 1.6 to 12.5 ₵/kWh[4]. The cost was further adjusted to 2018 dollars using the annual average chained consumer price index for all urban consumers[6].

Supplementary Note 3: Cost Allocation of Individual Subsystems at Hydrogen Production Plants

The NETL study[1] reports capital and O&M costs for hydrogen plants using coal and natural gas resources as feedstocks (Supplementary Table 1). In the NETL study[1], the total as-spent capital (TASC) is determined using total overnight cost (TOC) and TASC multiplier. The TOC of a hydrogen production plant includes total plant costs (TPCs) and owner's costs. However, the TPC is reported at a process or system level, whereas the owner's costs are reported at a plant level. In addition, the NETL study treats accessory electric plant, instrumentation & control, improvements to site, and buildings & structures as direct cost systems. The total O&M (TOM) cost includes fixed operating and maintenance (FOM), variable operating and maintenance (VOM) and fuel costs. CO_2 transportation and storage (CO_2T&S) cost is a standalone category. Below are the further discussions about cost components included in each type of cost categories[1]:

- As defined in the NETL report, individual process or system level TPCs are divided into 14 cost items (Supplementary Table 6). Each cost item includes components that are denoted as *Item No. x.xx* in the NETL study's 'total plant cost details' tables. TPC *Items No. 1–10* include the individual TPCs for specified processes or systems at a hydrogen production plant. TPC *Items No. 11–14* refers to accessory electric plant, instrumentation & control, improvements to site, and buildings & structures as direct cost systems, respectively.
- The owner's cost includes three subcategories: pre-production costs, inventory capital and other costs[1]. Each subcategory has various components in terms of relative labor, maintenance material, consumables, waste disposal, fuel, land, or financing costs. The cost of subcategories is estimated at the plant or system level.
- The FOM costs include annual operating labor, maintenance labor, administrative & support labor, property taxes and insurance.
- The VOM costs include maintenance material cost, consumables cost, and waste disposal cost. Consumables and waste disposal costs list consumables and waste disposal items.
- Fuel cost and CO_2T&S cost are categorized separately from the variable O&M costs.

Supplementary Table 6. Cost items included in total plant cost in the NETL study[1].

Item No.	Steam Methane Reforming with Carbon Capture and Storage	Coal Gasification with Carbon Capture and Storage
1	None	Coal Handling
2	None	Coal Preparation & Feed
3	Feedwater & Miscellaneous BOP Systems	Feedwater & Miscellaneous BOP Systems
4	Reformer & Accessories	Gasifier, ASU, & Accessories
5	Flue Gas Cleanup	Syngas Cleanup
6	Syngas Cleanup	Hydrogen Production
7	Hydrogen Production	Off Gas Fired Boiler, Ductwork, & Stack
8	Fired Heater, Ductwork, & Stack	Steam Turbine & Accessories
9	Cooling Water System	Cooling Water System
10	None	Slag Recovery & Handling
11	Accessory Electric Plant	Accessory Electric Plant
12	Instrumentation & Control	Instrumentation & Control
13	Improvements to Site	Improvements to Site
14	Buildings & Structures	Buildings & Structures

To construct capital and O&M learning curves for each subsystem defined in Supplementary Table 3 or Table 4, we have to determine the initial TASC and TOM of each subsystem installed at each type of hydrogen production plant. Thus, we have to make cost allocations to NETL's cost estimates to determine the TASC and TOM for the individual subsystems defined in Supplementary Tables 3–4. In general, there are two allocation methods: when costs are reported by NETL at the plant level, the relative share of TPCs within a subsystem to the total TPC for *Items No. 1 to 10* defined in Supplementary Table 6 is applied to make the cost allocation; when costs are reported by NETL at the system level, they are assigned to the corresponding subsystem based on the actual amount of resource use or waste discharge of each subsystem defined in Supplementary Tables 3 and 4. The cost allocation methods and results are discussed below.

The initial TASC is determined based on TOC and TASC multiplier. The TOC is estimated as the sum of TPCs and owner's cost for each subsystem defined in Supplementary Table 3 or Table 4. *Items No. 1–10* defined in Supplementary Table 6 are assigned to each corresponding subsystem defined in Supplementary Table 3 or Table 4. *Items No. 11–14* defined in Supplementary Table 6 are also assigned proportionally to *Items No. 1–10* based on the share of each item's TPC relative to the total TPC for *Items No. 1–10*.

The pre-production costs, inventory capital and other costs in the owner's cost include a variety of cost subcategories that report their cost estimations at the plant or system level. The cost reported by NETL at the plant level is allocated to individual subsystems based on the share of each item's TPC relative to the total TPC for *Items No. 1–10*, whereas those reported by NETL at the system level are assigned to the corresponding subsystem where they are actually consumed or discharged. The owner's cost allocation results are detailed in Supplementary Tables 7 and 8.

The initial TOM cost is estimated as the sum of FOM, VOM, fuel and CO_2T&S costs for each subsystem. In the NETL report, the FOM and maintenance material costs are reported at the plant level. As such, FOM and maintenance material costs are allocated to each subsystem based on the relative shares of TPCs for *Items No. 1–10*. In contrast, the VOM costs, such as consumables, waste disposal cost, and fuel and CO_2 T&S costs, are reported at the system level, except for the maintenance material cost. Hence, they are assigned to individual subsystems where they are actually used or discharged. However, if a resource, such as water or electricity, is consumed by multiple subsystems, that

consumables' cost is broken down to the multiple subsystems based on the actual amount of the resource consumed by each subsystem. Supplementary Tables 9 and 10 show the details of the TOM and CO_2T&S costs allocation results.

Supplementary Table 7. Cost categories included in owner's cost of steam methane reforming with carbon capture and storage in the NETL study[1].

Owner Cost Subcategories[1]			Allocation Basis	Allocated Subsystem(s)
Pre-Production Cost	6 Months All Labor		The Relative Shares of TPCs for *Items No. 1–10*	PSA, SMR, CO_2 Capture, CO_2 Compression
	1 Month Maintenance Materials			
	1 Month Non-Fuel Consumables	Water	The Actual Use of Each Subsystem	SMR, CO_2 Capture
		Makeup and Wastewater Treatment Chemicals		SMR
		Zinc Oxide Sulfur Guard Catalyst		SMR
		Prereformer Catalyst		SMR
		Primary Reformer Catalyst		SMR
		Water Gas Shift Catalyst		SMR
		Methyl Diethanolamine Solution		CO_2 Capture
		Post-Combustion CO_2 Capture		CO_2 Capture
		PSA Unit Adsorbent		PSA
		Triethylene Glycol		CO_2 Compression
		Electricity		PSA, SMR, CO_2 Capture, CO_2 Compression
	1 Month Waste Disposal	Zinc Oxide Sulfur Guard Catalyst	The Actual Waste Discharge of Each Subsystem	SMR
		Prereformer Catalyst		SMR
		Primary Reformer Catalyst		SMR
		Water Gas Shift Catalyst		SMR
		Methyl Diethanolamine Solution		CO_2 Capture
		PSA Unit Adsorbent		PSA
		Triethylene Glycol		CO_2 Compression
		Amine Purification Unit Waste		CO_2 Capture
		Thermal Reclaimer Unit Waste		CO_2 Capture
	25% of 1 Months Fuel Cost at 100% CF		The Actual Use of Each Subsystem	SMR

	2% of TPC		The Relative Shares of TPCs for *Items No. 1–10*	PSA, SMR, CO_2 Capture, CO_2 Compression
Inventory Capital	60-day Supply of Fuel and Consumables at 100% CF	Water	The Actual Use of Each Subsystem	SMR, CO_2 Capture
		Makeup and Wastewater Treatment Chemicals		SMR
		Zinc Oxide Sulfur Guard Catalyst		SMR
		Prereformer Catalyst		SMR
		Primary Reformer Catalyst		SMR
		Water Gas Shift Catalyst		SMR
		Methyl Diethanolamine Solution		CO_2 Capture
		Post-Combustion CO_2 Capture		CO_2 Capture
		PSA Unit Adsorbent		PSA
		Triethylene Glycol		CO_2 Compression
		Electricity		PSA, SMR, CO_2 Capture, CO_2 Compression
		Fuel		SMR
	0.5% of TPC (Spare Parts)		The Relative Shares of TPCs for *Items No. 1–10*	PSA, SMR, CO_2 Capture, CO_2 Compression
Other Costs	Initial Cost for Catalyst and Chemicals	Zinc Oxide Sulfur Guard Catalyst	The Actual Use of Each Subsystem	SMR
		Prereformer Catalyst		SMR
		Primary Reformer Catalyst		SMR
		Water Gas Shift Catalyst		SMR
		PSA Unit Adsorbent		PSA
		Methyl Diethanolamine Solution		CO_2 Capture
	Land		The Relative Shares of TPCs for *Items No. 1–10*	PSA, SMR, CO_2 Capture, CO_2 Compression
	Financing Costs			
	Other Owner's Costs			

Supplementary Table 8. Cost categories included in owner's cost of coal gasification with carbon capture and storage in the NETL study[1].

Owner Cost Subcategories[1]		Allocation Basis	Allocated Subsystem(s)
Pre-Production Cost	6 Months All Labor	The Relative Shares of TPCs for *Items No. 1–10*	ASU, Gasification Block, Syngas Cleanup, Sulfur
	1 Month Maintenance Materials		

				Recovery, PSA, Power Block, CO_2 Capture, CO_2 Compression
	1 Month Non-Fuel Consumables	Water	The Actual Use of Each Subsystem	Syngas Cleanup, Power Block
		Makeup and Wastewater Treatment Chemicals		Power Block
		Sulfur-Impregnated Activated Carbon		Syngas Cleanup
		Water Gas Shift Catalyst		Syngas Cleanup
		Selexol Solution		CO_2 Capture
		Sodium Hydroxide (50 wt.%)		Syngas Cleanup
		Sulfuric Acid (98 wt.%)		Syngas Cleanup
		Claus Catalyst		Sulfur Recovery
		PSA Unit Adsorbent		PSA
		Triethylene Glycol		CO_2 Compression
		Electricity		ASU, Gasification Block, Syngas Cleanup, Sulfur Recovery, PSA, Power Block, CO_2 Capture, CO_2 Compression
	1 Month Waste Disposal	Sulfur-Impregnated Activated Carbon	The Actual Waste Discharge of Each Subsystem	Syngas Cleanup
		Water Gas Shift Catalyst		Syngas Cleanup
		Selexol Solution		CO_2 Capture
		Claus Catalyst		Sulfur Recovery
		Crystallizer Solids		Power Block
		Slag		Gasification Block
		PSA Unit Adsorbent		PSA
		Triethylene Glycol		CO_2 Compression
	25% of 1 Months Fuel Cost at 100% CF		The Actual Use of Each Subsystem	Gasification Block
	2% of TPC		The Relative Shares of TPCs for *Items No. 1–10*	ASU, Gasification Block, Syngas Cleanup, Sulfur Recovery, PSA, Power Block, CO_2 Capture,

				CO_2 Compression
Inventory Capital	60-day Supply of Fuel and Consumables at 100% CF	Water	The Actual Use of Each Subsystem	Syngas Cleanup, Power Block
		Makeup and Wastewater Treatment Chemicals		Power Block
		Sulfur-Impregnated Activated Carbon		Syngas Cleanup
		Water Gas Shift Catalyst		Syngas Cleanup
		Selexol Solution		CO_2 Capture
		Sodium Hydroxide (50 wt.%)		Syngas Cleanup
		Sulfuric Acid (98 wt.%)		Syngas Cleanup
		Claus Catalyst		Sulfur Recovery
		PSA Unit Adsorbent		PSA
		Triethylene Glycol		CO_2 Compression
		Electricity		ASU, Gasification Block, Syngas Cleanup, Sulfur Recovery, PSA, Power Block, CO_2 Capture, CO_2 Compression
		Fuel		Gasification Block
	0.5% of TPC (Spare Parts)		The Relative Shares of TPCs for *Items No. 1–10*	ASU, Gasification Block, Syngas Cleanup, Sulfur Recovery, PSA, Power Block, CO_2 Capture, CO_2 Compression
Other Costs	Initial Cost for Catalyst and Chemicals	Sulfur-Impregnated Activated Carbon	The Actual Use of Each Subsystem	Syngas Cleanup
		Water Gas Shift Catalyst		Syngas Cleanup
		Selexol Solution		CO_2 Capture
		PSA Unit Adsorbent		PSA
	Land		The Relative Shares of TPCs for *Items No. 1–10*	ASU, Gasification Block, Syngas Cleanup, Sulfur Recovery, PSA, Power Block, CO_2 Capture,
	Financing Costs			
	Other Owner’s Costs			

			CO_2 Compression

Supplementary Table 9. Cost categories included in FOM, VOM, fuel and CO_2T&S costs of steam methane reforming with carbon capture and storage from the NETL study[1].

O&M Components			Allocation Basis	Allocated Subsystem(s)
FOM Costs	Annual Operating Labor		The Relative Shares of TPCs for *Items No. 1–10*	PSA, SMR, CO_2 Capture, CO_2 Compression
	Maintenance Labor			
	Administrative & Support Labor			
	Property Taxes and Insurance			
VOM Costs	Maintenance Material		The Relative Shares of TPCs for *Items No. 1–10*	PSA, SMR, CO_2 Capture, CO_2 Compression
	Consumables	Water	The Actual Use of Each Subsystem	SMR, CO_2 Capture
		Makeup and Wastewater Treatment Chemicals		SMR
		Zinc Oxide Sulfur Guard Catalyst		SMR
		Prereformer Catalyst		SMR
		Primary Reformer Catalyst		SMR
		Water Gas Shift Catalyst		SMR
		Methyl Diethanolamine Solution		CO_2 Capture
		Post-Combustion CO_2 Capture		CO_2 Capture
		PSA Unit Adsorbent		PSA
		Triethylene Glycol		CO_2 Compression
		Electricity		PSA, SMR, CO_2 Capture, CO_2 Compression
	Waste Disposal	Zinc Oxide Sulfur Guard Catalyst	The Actual Waste Discharge of Each Subsystem	SMR
		Prereformer Catalyst		SMR
		Primary Reformer Catalyst		SMR
		Water Gas Shift Catalyst		SMR
		Methyl Diethanolamine Solution		CO_2 Capture
		PSA Unit Adsorbent		PSA
		Triethylene Glycol		CO_2 Compression
		Amine Purification Unit Waste		CO_2 Capture

		Thermal Reclaimer Unit Waste		CO_2 Capture
Fuel		Natural Gas	The Actual Use of Each Subsystem	SMR
CO_2T&S Cost				CO_2 Transportation and Storage

Supplementary Table 10. Cost categories included in FOM, VOM, fuel and CO_2T&S costs of coal gasification with carbon capture and storage from the NETL study[1].

**O&M Components**			**Allocation Basis**	**Allocated Subsystem(s)**
FOM Costs	Annual Operating Labor		The Relative Shares of TPCs for *Items No. 1–10*	ASU, Gasification Block, Syngas Cleanup, Sulfur Recovery, PSA, Power Block, CO_2 Capture, CO_2 Compression
	Maintenance Labor			
	Administrative & Support Labor			
	Property Taxes and Insurance			
VOM Costs	Maintenance Material		The Relative Shares of TPCs for *Items No. 1–10*	ASU, Gasification Block, Syngas Cleanup, Sulfur Recovery, PSA, Power Block, CO_2 Capture, CO_2 Compression
	Consumables	Water	The Actual Use of Each Subsystem	Syngas Cleanup, Power Block
		Makeup and Wastewater Treatment Chemicals		Power Block
		Sulfur-Impregnated Activated Carbon		Syngas Cleanup
		Water Gas Shift Catalyst		Syngas Cleanup
		Selexol Solution		CO_2 Capture
		Sodium Hydroxide (50 wt.%)		Syngas Cleanup
		Sulfuric Acid (98 wt.%)		Syngas Cleanup
		Claus Catalyst		Sulfur Recovery
		PSA Unit Adsorbent		PSA
		Triethylene Glycol		CO_2 Compression
		Electricity		ASU, Gasification Block, Syngas Cleanup, Sulfur Recovery, PSA, Power Block, CO_2 Capture, CO_2 Compression
	Waste Disposal	Sulfur-Impregnated Activated Carbon	The Actual Waste Discharge of Each Subsystem	Syngas Cleanup
		Water Gas Shift Catalyst		Syngas Cleanup
		Selexol Solution		CO_2 Capture

		Claus Catalyst		Sulfur Recovery
		Crystallizer Solids		Power Block
		Slag		Gasification Block
		PSA Unit Adsorbent		PSA
		Triethylene Glycol		CO_2 Compression
Fuel		Illinois No. 6 Coal	The Actual Use of Each Subsystem	Gasification Block
CO_2T&S Cost				CO_2 Transportation and Storage

Supplementary Note 4: Costs and Initial Installed Capacity of Individual Subsystems at Clean Hydrogen Production Plants

The initial installed capacity data collected from the literature are reported on inconsistent bases for individual subsystems, which are on a mass basis or an electric power basis. To harmonize inconsistent metrics, the learning curves in this study are initially developed on a thermal energy basis for hydrogen and then converted to those on a mass basis. Specifically, the initial learning curves report thermal-based cumulative installed capacity (GW_{th}) in the x-axis and dollars per thermal-equivalent megawatt and megawatt hour for capital and O&M costs in the y-axis, respectively. To develop a mass-based learning curve, the thermal-based cumulative installed capacity and costs are then converted to the mass-based measures using the method discussed below.

First, the initial costs on an absolute basis (Supplementary Tables 11 and 12) and initial installed capacity of each subsystem on mass-basis or electric power-based (Supplementary Table 13) are converted to thermal-based values. To convert the mass-based or electric power-based measure to the thermal-based measure, the higher heating value (HHV) of hydrogen[7] or the plant efficiency of a modern power plant is used. The HHV of hydrogen used in this study is 39.41 kWh/kg[7]. Referring to an integrated coal gasification combined cycle plant or a supercritical coal plant, a plant efficiency of 40% is assumed. When the raw data of initial installed capacity are reported on a mass basis, the thermal-equivalent installed capacity of a subsystem is calculated using Supplementary Equations (2) and (3). When the raw data of initial installed capacity are reported on an electric power basis, the thermal-equivalent installed capacity of a subsystem is calculated using Supplementary Equation (4).

$$\mathrm{EKG_{H2}} = \frac{\mathrm{EKG_{H2}^{year}} \cdot \mathrm{C_{f1}}}{\mathrm{AH} \cdot \mathrm{CF}} \quad \text{(S2)}$$

$$\mathrm{MW_{th}} = \frac{\alpha \cdot \mathrm{EKG_{H2}}}{\mathrm{C_{f2}}} \quad \text{(S3)}$$

$$\mathrm{MW_{th}} = \frac{\mathrm{MW_e}}{\mathrm{E_f}} \quad \text{(S4)}$$

Where EKG_{H2} is the estimated hourly initial installed hydrogen production rate (kg H_2/hour); $\mathrm{EKG_{H2}^{year}}$ is the estimated initial installed hydrogen production capacity given in Supplementary Table 13 (metric ton/year); C_{f1} is the unit conversion 1000 (kg/metric ton); AH is the total annual hours (8760 hours/year); CF is the plant capacity factor (%); MW_{th} is the thermal energy-based hydrogen production capacity (MW_{th}); α is the higher heating value of hydrogen (39.41 kWh/kg)[7]; C_{f2} is the unit conversion 1000 (kW/MW); MW_e is the collected electric power-based initial installed capacity given in Supplementary Table 13 (MW_e); and E_f is the assumed 40% efficiency of an integrated coal gasification combined cycle plant or a supercritical coal plant (%).

The capital and O&M costs on an absolute basis are respectively converted to the thermal energy-based costs of $/$MW_{th}$ (capital costs) and $/$MWh_{th}$ (O&M costs) in terms of hydrogen production capacity and

HHV. The thermal energy-based initial capital and O&M costs are estimated using the following supplementary equations, respectively:

$$\mathrm{TASC_2} = \frac{\mathrm{TASC_1} \cdot 1000}{\mathrm{KG_{H2}}} \tag{S5}$$

$$\mathrm{TASC_3} = \frac{\mathrm{TASC_2} \cdot \mathrm{C_{f2}}}{\alpha} \tag{S6}$$

$$\mathrm{TOM_2} = \frac{\mathrm{TOM_1}}{\mathrm{AHP_{H2}}} \tag{S7}$$

$$\mathrm{TOM_3} = \frac{\mathrm{TOM_2} \cdot \mathrm{C_{f2}}}{\alpha} \tag{S8}$$

Where $TASC_1$ is the initial capital cost on an absolute basis ($/1000); $TASC_2$ is the normalized initial capital cost ($/kg H_2/hour); $\mathrm{KG_{H2}}$ is the hourly hydrogen production rate (kg H_2/hour); $TASC_3$ is the thermal energy-based initial capital cost ($/$MW_{th}$); TOM_1 is the initial O&M costs on an absolute basis ($/year); TOM_2 is the normalized initial O&M cost ($/kg H_2); $\mathrm{AHP_{H2}}$ is the annual hydrogen production (kg H_2/year); TOM_3 is the thermal energy-based initial O&M cost ($/$MWh_{th}$); C_{f2} and α are defined as in Supplementary Equation (3).

The thermal-based future capital and O&M cost estimates by learning curves are converted to the mass-based cost estimates using Supplementary Equations (9) and (10), respectively. In addition, the cumulative installed capacity of future hydrogen production is converted from the thermal-equivalent estimate to the mass-based estimate using Supplementary Equation (11).

$$\mathrm{TASC_{f2}} = \frac{\mathrm{TASC_{f1}} \cdot \alpha \cdot \mathrm{KG_{H2}}}{\mathrm{C_{f2}}} \tag{S9}$$

$$\mathrm{TOM_{f2}} = \frac{\mathrm{TOM_{f1}} \cdot \alpha \cdot \mathrm{AHP_{H2}}}{\mathrm{C_{f2}}} \tag{S10}$$

$$\mathrm{FKG_{H2}^{year}} = \frac{\mathrm{FMW_{th}} \cdot \mathrm{C_{f2}} \cdot \mathrm{AH} \cdot \mathrm{CF}}{\alpha \cdot \mathrm{C_{f1}}} \tag{S11}$$

Where $TASC_{f1}$ is the projected thermal energy-based future hydrogen production capital cost ($/$MW_{th}$); $TASC_{f2}$ is the projected mass-based future hydrogen production capital cost ($); TOM_{f1} is the projected thermal energy-based future hydrogen production O&M cost ($/$MWh_{th}$); TOM_{f2} is the projected mass-based future hydrogen production O&M cost ($/year); FMW_{th} is the thermal-based future cumulative installed hydrogen production capacity (MW_{th}); $\mathrm{FKG_{H2}^{year}}$ is the mass-based future cumulative installed hydrogen production capacity (metric ton/year); α, AH, CF, C_{f1}, C_{f2}, $\mathrm{KG_{H2}}$ and $\mathrm{AHP_{H2}}$ are defined as in Supplementary Equations (2), (3), (5) and (7), respectively.

Supplementary Table 11. Initial capital and O&M costs of subsystems for steam methane reforming with carbon capture outlined in the NETL study[1].

Subsystem	Total As-Spent Capital ($/1000)	Total Normalized Capital cost ($/kg H_2/hour)[a]	Total Normalized Capital cost ($/$MW_{th}$)[b]	Annual Total O&M Cost ($/year)	Total Normalized O&M cost ($/kg H_2)[c]	Total Normalized O&M cost ($/$MWh_{th}$)[d]
PSA	58,472	2,905	73,722	7,988,396	0.05	1.28
SMR	343,477	17,067	433,061	150,448,795	0.95	24.06
CO_2 Capture	383,946	19,078	484,085	19,919,630	0.13	3.19
CO_2 Compression	110,767	5,504	139,657	13,735,136	0.09	2.20

CO_2 Transportation and Storage	0	0	0	15,169,841	0.10	2.43

[a]The value is calculated using Supplementary Equation (5).
[b]The value is calculated using Supplementary Equation (6).
[c]The value is calculated using Supplementary Equation (7).
[d]The value is calculated using Supplementary Equation (8).

Supplementary Table 12. Initial capital and O&M costs of subsystems for coal gasification with carbon capture and storage outlined in the NETL study[1].

Subsystem	Total As-Spent Capital ($/1000)	Total Normalized Capital cost ($/kg H_2/hour)[a]	Total Normalized Capital cost ($/$MW_{th}$)[b]	Annual Total O&M Cost ($/year)	Total Normalized O&M cost ($/kg H_2)[c]	Total Normalized O&M cost ($/$MWh_{th}$)[d]
ASU	389,034	14,147	358,959	25,802,111	0.13	3.40
Gasification Block	2,199,820	79,993	2,029,764	181,015,126	0.94	23.83
Syngas Cleanup	510,400	18,560	470,944	27,340,312	0.14	3.60
Sulfur Recovery	229,686	8,352	211,930	9,644,838	0.05	1.27
PSA	62,332	2,267	57,514	3,931,253	0.02	0.52
Power Block	385,863	14,031	356,034	20,127,732	0.10	2.65
CO_2 Capture	573,416	20,851	529,088	24,693,539	0.13	3.25
CO_2 Compression	102,423	3,724	94,506	9,814,366	0.05	1.29
CO_2 Transportation and Storage	0	0	0	31,981,989	0.17	4.21

[a]The value is calculated using Supplementary Equation (5).
[b]The value is calculated using Supplementary Equation (6).
[c]The value is calculated using Supplementary Equation (7).
[d]The value is calculated using Supplementary Equation (8).

Supplementary Table 13. Initial installed capacity of individual subsystems.

Feedstock	Subsystems	Initial Installed Hydrogen Production Capacity (million metric tons/year)[a]	Initial Installed Capacity (GW_e)[b]	Initial Installed Capacity (GW_{th})
Natural Gas	SMR	56.0	Not Applicable	279.7[c]
	PSA	73.2	Not Applicable	365.7[c]
	CO_2 Capture	0.31	Not Applicable	1.5[c]
	CO_2 Compression	Not Applicable	10	25.0[d]
	CO_2 Transportation and Storage	0.46[f]	Not Applicable	2.4[g]
Coal	ASU	Not Applicable	50	125.0[d]
	Gasification Block	Not Applicable	Not Applicable	173.0[e]

	Syngas Cleanup	Not Applicable	Not Applicable	173.0[e]
	Sulfur Recovery	Not Applicable	50	125.0[d]
	PSA	73.2	Not Applicable	411.5[c]
	Power Block	Not Applicable	240	600.0[d]
	CO_2 Capture	0.15	Not Applicable	0.9[c]
	CO_2 Compression	Not Applicable	10	25.0[d]
	CO_2 Transportation and Storage	0.46[f]	Not Applicable	2.4[g]

[a]The initial installed capacity of hydrogen production is collected from the International Energy Agency (IEA) studies (2021 and 2022)[8,9], and is converted to thermal energy based initial installed capacity using Supplementary Equations (2) and (3).
[b]The initial installed capacities of these subsystems are collected from historical data (Rubin et al., 2007)[10], and will be converted to thermal energy based initial installed capacity using Supplementary Equation (4).
[c]The thermal energy based initial installed capacity is calculated based on Supplementary Equations (2) and (3).
[d]The thermal energy based initial installed capacity is calculated based on Supplementary Equation (4).
[e]The thermal energy based initial installed capacity is obtained from Higman (2017)[11].
[f]This is a common subsystem for both the gas- and coal-based hydrogen production plants. The value is the summation of mass-based capacity of CO_2 transportation and storage subsystems from gas- and coal-based hydrogen production.
[g]This number is the summation of thermal energy-based capacity of CO_2 transportation and storage subsystems of gas- and coal-based hydrogen production. The capacity factor is assumed to be 90% for the gas-based hydrogen production and 80% for the coal-based hydrogen production when estimating the thermal energy-based capacity[1].

Supplementary Note 5: Fossil Fuel-Based Hydrogen Production Life Cycle Emissions and Tax Credits

Supplementary Note 5 summarizes the life cycle emissions for two hydrogen production plants, as displayed in Supplementary Table 14. These emissions data are retrieved from the NETL study (2022)[1].

This note also presents two tax incentives (i.e., Sections 45V and 45Q) outlined in the Inflation Reduction Act[12]. As shown in Supplementary Table 15, the Section 45V tax credit is dependent on life cycle greenhouse emissions from hydrogen production. Its bonus tax credit ranges from \$0.6–3.0/kg H_2 if prevailing wage and apprenticeship requirements are met. As shown in Supplementary Table 16, the Section 45Q tax credit is offered to either carbon sequestration by geological storage or utilization. It has a bonus tax credit of \$85/metric ton CO_2 for carbon dioxide stored in saline reservoirs when meeting the prevailing wage and apprenticeship requirements. In this study, when referring to the 45Q tax credit, it represents an incentive tax credit of \$85 per metric ton of CO_2 stored in saline reservoirs; and hydrogen production facilities are assumed to be eligible for the tax credit of Section 45V.

Supplementary Fig. 3 displays the effects of tax credit period on future cost of gas-based blue hydrogen production.

Supplementary Table 14. Life cycle greenhouse gas emissions for gas-based and coal-based blue hydrogen production plants[1].

Technology		Steam Methane Reforming with Carbon Capture and Storage	Coal Gasification with Carbon Capture and Storage
LCA GHG Emissions (kg CO_2-eq/kg H_2)	Stack Emission	0.38	1.35
	Natural Gas Emission	2.86	N/A
	Coal Emission	N/A	1.63
	Grid Electricity Emission	1.19	0.83
	CO_2 Management Emission	0.15	0.26
	Total	4.57	4.08
	Low (5th Percentile)	3.13	3.40
	High (95th Percentile)	8.86	8.87

Supplementary Table 15. Section 45V tax credit[12].

Life Cycle Greenhouse Gas Emissions Rate (kg CO_2-eq/kg H_2)	45V Bonus Rate ($/kg H_2)
< 0.45	3.00
< 1.5 and ≥ 0.45	1.00
< 2.5 and ≥ 1.5	0.75
< 4.0 and ≥ 2.5	0.60

Supplementary Table 16. Section 45Q tax credit[12].

CO_2 Sequestration or Utilization	45Q Bonus Rate ($/metric ton CO_2)
CO_2 Stored in Saline Reservoirs	85
CO_2 Sale for Utilization	60

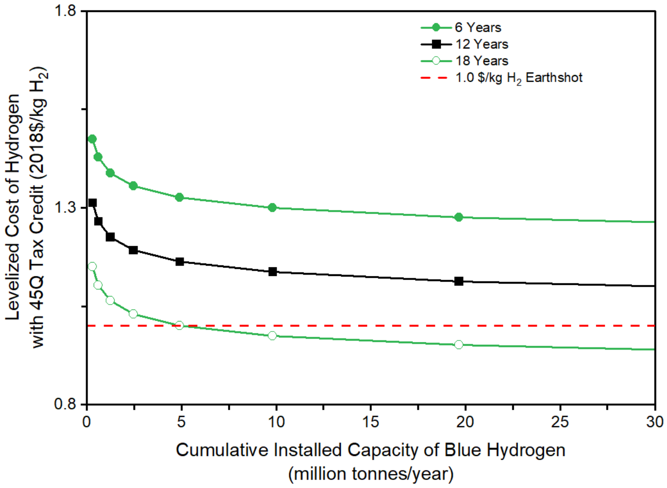

Supplementary Fig. 3. Effect of 45Q tax credit period on future cost of gas-based blue hydrogen production.

Supplementary Note 6: Natural Resource Consumption Intensities of Gas-based Blue Hydrogen

This note shows the natural resource consumption as a function of cumulative installed capacity of blue hydrogen, such as natural gas resources, land resources, and water withdrawals for SMR-CCS.

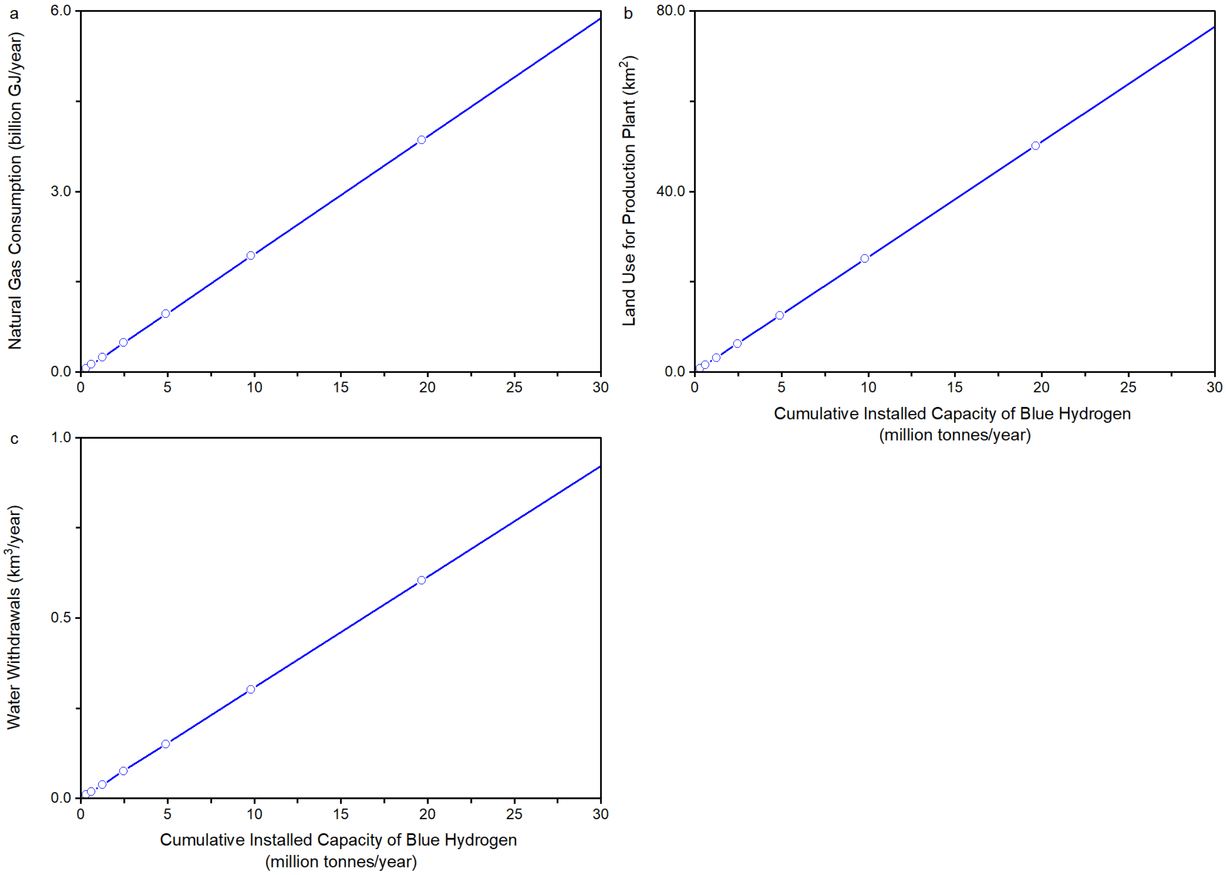

Supplementary Fig. 4. Natural resource requirements for gas-based blue hydrogen production as a function of cumulative installed hydrogen production capacity. (**a**) Natural gas resources; (**b**) Land resources; (**c**) Water withdrawals.

Supplementary Note 7: Development of a Time-based Diffusion Model of Low-Carbon Hydrogen

To estimate the cumulative installed capacity over time, a diffusion-of-innovation model is developed based on the current and future hydrogen capacity. The scatter points shown in Supplementary Fig. 5a represent the installed capacity of global fossil fuels with CCUS for low-carbon hydrogen production in 2021 and the cumulative installed capacity of low-carbon production in 2024–2030. The data are retrieved from the IEA studies[8,9] and a study of the Hydrogen Council and McKinsey & Company[13], respectively. The fitting curve in Supplementary Fig. 5a is the nonlinear fitting result of the scatter points. This fitting curve is used to calculate the annual installed capacity of low-carbon hydrogen from 2021 to 2029, as shown in Supplementary Fig. 5b. The annual installed capacity of low-carbon hydrogen is used for the fitting of the diffusion model. Supplementary Fig. 6 shows the diffusion model and the annual installed

capacity of global low-carbon hydrogen through 2050.

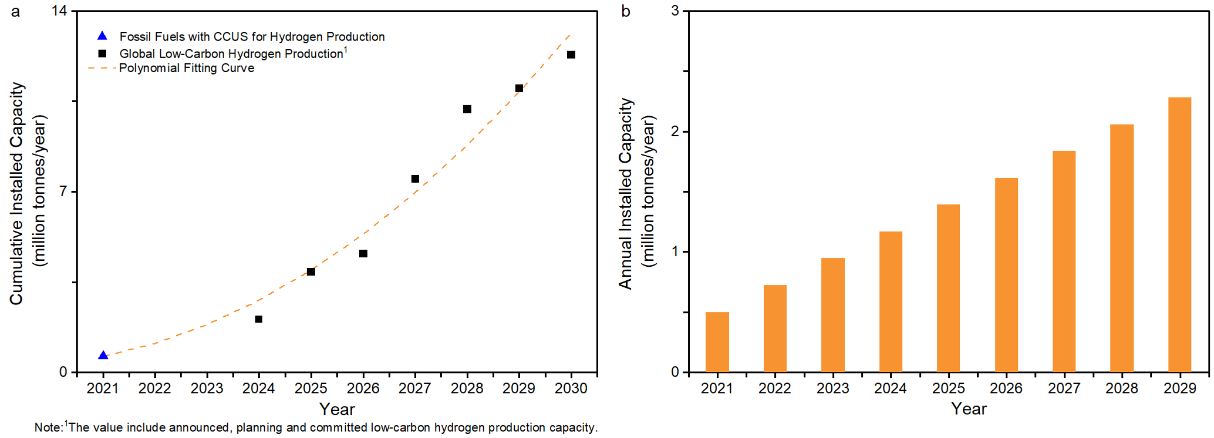

Supplementary Fig. 5. Cumulative and annual installed capacity of global low-carbon hydrogen over time. (**a**) Cumulative installed capacity; (**b**) Annual installed capacity.

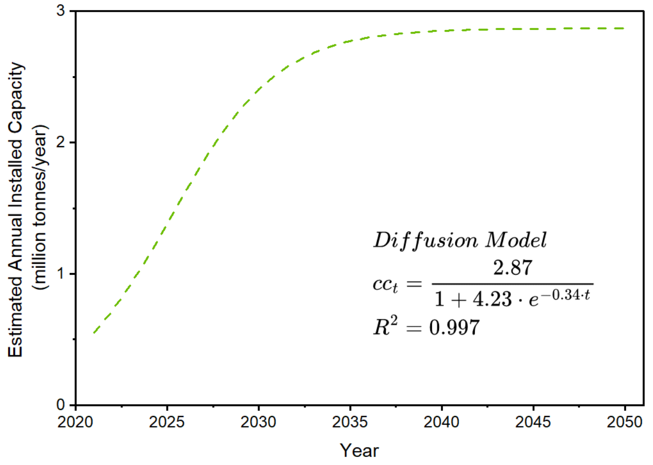

Supplementary Fig. 6. Projection of annual installed capacity of global low-carbon hydrogen through 2050.

Supplementary Note 8: Hydrogen Production Cost Estimation in Nominal Dollars

The levelized cost of hydrogen is estimated in nominal dollars[1]:

$$\mathrm{LCOH_N = LCC_N + LOM_N + LFP_N} \quad \text{(S12)}$$

$$\mathrm{LCOH_{N,TC} = LCC_N + LOM_N + LFP_N - LTC_{N,45Q}} \quad \text{(S13)}$$

Where the subscript “N” means the nominal dollars; $LCOH_N$ is the levelized cost of hydrogen (\$/kg H_2); LCC_N is the levelized capital cost (\$/kg H_2); LOM_N is the levelized operating and maintenance cost (\$/kg H_2); LFP_N is the levelized fuel price (\$/kg H_2); $\mathrm{LCOH_{N,TC}}$ is the levelized cost of hydrogen with a tax credit (\$/kg H_2); $\mathrm{LTC_{N,45Q}}$ is the levelized tax credit of Section 45Q over the project book lifetime of a blue hydrogen project (\$/kg H_2).

The levelized capital cost in nominal dollars is estimated using Supplementary Equation (14), while the fixed charge rate is estimated using Supplementary Equations (15) to (21)[2,3,14]:

$$\mathrm{LCC_N = \frac{TASC_R \cdot FCR_N}{(CF \cdot AH) \cdot KG_{H2}}} \quad \text{(S14)}$$

$$\mathrm{FCR_N} = \frac{\mathrm{CRF_N^{nonfuel}} - \mathrm{ETR} \cdot \mathrm{PV_{plant}}}{1 - \mathrm{ETR}} \tag{S15}$$

$$\mathrm{CRF_N^{nonfuel}} = \frac{\mathrm{ATWACC_N} \cdot (1 + \mathrm{ATWACC_N})^{\mathrm{BL}}}{(1 + \mathrm{ATWACC_N})^{\mathrm{BL}} - 1} \tag{S16}$$

$$\mathrm{PV_{plant}} = \mathrm{CRF_N^{nonfuel}} \cdot \sum_{\mathrm{n}=1}^{\mathrm{m}} \frac{\mathrm{d_n}}{(1 + \mathrm{ATWACC_N})^{\mathrm{n}}} \tag{S17}$$

$$\mathrm{ATWACC_N} = \mathrm{PC_{equity}} \cdot \mathrm{ROE_N} + \mathrm{PC_{debt}} \cdot \mathrm{kd_N} \cdot (1 - \mathrm{ETR}) \tag{S18}$$

$$\mathrm{ROE_N} = (1 + \mathrm{ROE_R}) \cdot (1 + N) - 1 \tag{S19}$$

$$\mathrm{kd_N} = (1 + \mathrm{kd_R}) \cdot (1 + N) - 1 \tag{S20}$$

$$N = R + I \tag{S21}$$

Where the superscript "nonfuel" represents the non-fuel component; $TASC_R$ is the total as-spent capital of a blue hydrogen production plant ($); FCR_N is the fixed charge rate (fraction/year); CF is the plant capacity factor (%); AH is the total annual hours (8760 hours); KG_{H2} is the hourly hydrogen production rate (kg H_2/hour); CRF_N is the capital recovery factor (fraction/year); ETR is the effective tax rate (%); PV_{plant} is the present value of tax depreciation expense (fraction/year); $ATWACC_N$ is the after-tax weighted average cost of capital (%); BL is the project book lifetime (year); d_n is the tax depreciation fraction in year n (fraction); m is the number of years of depreciation (year); PC_{equity} is the percent of equity (%); ROE_N is the nominal rate of return on equity (%); PC_{debt} is the percent of debt (%); kd_N is the nominal rate of cost of debt (%); ROE_R is the real rate of return on equity (%); kd_R is the real rate of cost of debt (%); N is the nominal escalation rate (%); R is the real escalation rate (%); and I is the inflation rate (%).

The levelized operating and maintenance cost in nominal dollars is estimated using Supplementary Equation (22), while the levelization factor is estimated using Supplementary Equation (23)[3]:

$$\mathrm{LOM_N} = \frac{\mathrm{OM_R} \cdot \mathrm{LF_N}}{(\mathrm{CF} \cdot \mathrm{AH}) \cdot \mathrm{KG_{H2}}} \tag{S22}$$

$$\mathrm{LF_N} = \mathrm{CRF_N^{nonfuel}} \cdot \frac{1 - \left[\frac{1 + N}{1 + \mathrm{ATWACC_N}}\right]^{\mathrm{BL}}}{\mathrm{ATWACC_N} - N} \tag{S23}$$

Where OM_R is the non-fuel O&M cost of a blue hydrogen production plant ($/year); and LF_N is the levelization factor for non-fuel O&M cost (unitless); LOM_N, CF, AH, CRF_N, $ATWACC_N$, BL and N are defined as in Supplementary Equations (12), (14), (15), (16), and (21), respectively.

The levelized fuel price in nominal dollars is estimated using Supplementary Equation (24), where the fuel's capital recovery factor is estimated using Supplementary Equation (26) and the discount rate in nominal dollars is estimated using Supplementary Equation (27)[15]:

$$\mathrm{LFP_N} = \frac{\mathrm{FC_N} \cdot \mathrm{FR}}{\mathrm{KG_{H2}}} \tag{S24}$$

$$\mathrm{FC_N} = \mathrm{PV_{fuel}} \cdot \mathrm{CRF_N^{fuel}} \tag{S25}$$

$$\mathrm{CRF_N^{fuel}} = \frac{\mathrm{DR_N} \cdot (1 + \mathrm{DR_N})^{\mathrm{BL}}}{(1 + \mathrm{DR_N})^{\mathrm{BL}} - 1} \tag{S26}$$

$$\mathrm{DR_N} = (1 + \mathrm{DR_R}) \cdot (1 + N) - 1 \tag{S27}$$

$$\mathrm{PV_{fuel}} = \sum_{\mathrm{t}=1}^{\mathrm{BL}} \frac{P_{\mathrm{t}}}{(1 + \mathrm{DR_N})^{\mathrm{t}}} \tag{S28}$$

$$P_{\mathrm{t}} = P_{\mathrm{t-1}} \cdot (1 + N) \tag{S29}$$

Where the superscript "fuel" represents the fuel component; FC_N is the natural gas cost ($/GJ);

FR is the hourly natural gas flow rate (GJ/hour); PV_{fuel} is the present value of gas cost ($/GJ); CRF_N^{fuel} is the fuel's capital recovery factor (fraction/year); DR_N is the nominal discount rate for fuel cost (%); DR_R is the real discount rate for fuel cost (%); N is the nominal escalation rate (%); P_t is the fuel cost in year t ($/GJ), while P_1 is the real fuel cost in the first year of a project; LFP_N, KG_{H2}, and BL are defined as in Supplementary Equations (12), (14), and (16), respectively.

The levelized tax credit is estimated in nominal dollars using Supplementary Equation (30):

$$\mathrm{LTC}_{\mathrm{N,45Q}} = \frac{\mathrm{TC}_{\mathrm{45Q}} \cdot \mathrm{ACS}_{\mathrm{CO2}} \cdot \mathrm{CP}_{\mathrm{45Q}}}{\mathrm{BL} \cdot \mathrm{AHP}_{\mathrm{H2}}} \cdot \mathrm{LF}_{\mathrm{N}} \tag{S30}$$

Where TC_{45Q} is the 45Q bonus rate ($/metric ton CO_2); ACS_{CO2} is the annual CO_2 sequestration amount (metric ton CO_2/year); CP_{45Q} is the 45Q credit period (years); AHP_{H2}, and $LTC_{N,45Q}$, LF_N, BL are defined as in Supplementary Equations (7), (13), (16), and (23), respectively.

In this study, the financial structure and assumptions are in alignment with the studies of the NETL[1,2,3,15] and the report of Internal Revenue Service (IRS)[16]. Supplementary Table 17 lists the financial parameters and summarizes their data and the sources of data. Supplementary Table 18 summarizes the estimates of FCR, levelization factor, and discount rate as a function of nominal escalation rate.

Supplementary Table 17. Financial parameters and assumptions

Parameter	Symbol	Unit	Value	Source(s)
Effective Tax Rate	ETR	%	25.74%	NETL (2021)[3]
Inflation Rate	I	%	0–3%	Assumption
Real Escalation Rate	R	%	0%	NETL (2011)[2]
Percent of Equity	PC_{equity}	%	62%	NETL (2022)[1]
Percent of Debt	PC_{debt}	%	38%	NETL (2022)[1]
Real Rate of Return on Equity	ROE_R	%	3.10%	NETL (2022)[1]
Real Rate of Cost of Debt	kd_R	%	5.15%	NETL (2022)[1]
Real Discount Rate	DR_R	%	4.73%	NETL (2019)[15]
Project Book Lifetime	BL	year	30	NETL (2022)[1]
Fuel Cost	P_1	2018$/GJ	4.2	NETL (2022)[1]
Tax Depreciation Fraction	d_1	%	3.75%	NETL (2021)[3]; IRS (2016)[16]
	d_2		7.22%	
	d_3		6.68%	
	d_4		6.18%	
	d_5		5.71%	
	d_6		5.29%	
	d_7		4.89%	
	d_8		4.52%	
	d_9		4.46%	
	d_{10}		4.46%	
	d_{11}		4.46%	
	d_{12}		4.46%	
	d_{13}		4.46%	
	d_{14}		4.46%	
	d_{15}		4.46%	
	d_{16}		4.46%	
	d_{17}		4.46%	

	d_{18}		4.46%	
	d_{19}		4.46%	
	d_{20}		4.46%	
	d_{21}		2.23%	

Supplementary Table 18. Estimates of fixed charge rate, levelization factor, and discount rate.

**Parameter**	**Symbol**	**Unit**	**Nominal Escalation Rate**			
			0%	1%	2%	3%
Fixed Charge Rate	FCR	fraction/year	0.059	0.067	0.076	0.085
Levelization Factor	LF	fraction	1.000	1.124	1.256	1.393
Discount Rate	DR	%	4.73%	5.78%	6.82%	7.87%

Supplementary References

1. National Energy Technology Laboratory. *Comparison of commercial, state-of-the-art, fossil-based hydrogen production technologies.* Report No. DOE/NETL-2022/3241 (2022).
2. National Energy Technology Laboratory. *Quality guidelines for energy system studies: cost estimation methodology for NETL assessments of power plant performance.* Report No. DOE/NETL-2011/1455 (2011).
3. National Energy Technology Laboratory. *Quality guidelines for energy system studies: cost estimation methodology for NETL assessments of power plant performance.* Report No. NETL-PUB-22580 (2021).
4. Peterson, D., Vickers, J. & DeSantis, D. *Hydrogen Production Cost from PEM Electrolysis-2019*. Prepared for Department of Energy. https://www.hydrogen.energy.gov/docs/hydrogenprogramlibraries/pdfs/19009_h2_production_cost_pem_electrolysis_2019.pdf?Status=Master (2020).
5. Palmer, G., Roberts, A., Hoadley, A., Dargaville, R. & Honnery, D. Life-cycle greenhouse gas emissions and net energy assessment of large-scale hydrogen production via electrolysis and solar PV. *Energy Environ. Sci.* **14**, 5113–5131 (2021).
6. U.S. Bureau of Labor Statics. *Chained consumer price index for all urban consumers (C-CPI-U): U.S. city average*. https://data.bls.gov/PDQWeb/su (2023).
7. Schoots, K., Ferioli, F., Kramer, G.J. & Van der Zwaan, B.C.C. Learning curves for hydrogen production technology: an assessment of observed cost reductions. *Int. J. Hydrog. Energy* **33**, 2630–2645 (2008).
8. International Energy Agency. *Hydrogen projects database*. https://www.iea.org/reports/hydrogen-projects-database (2021)
9. International Energy Agency. *Global hydrogen review 2022.* https://iea.blob.core.windows.net/assets/c5bc75b1-9e4d-460d-9056-6e8e626a11c4/GlobalHydrogenReview2022.pdf (2022).
10. Rubin, E.S., Yeh, S., Antes, M., Berkenpas, M. & Davison, J. Use of experience curves to estimate the future cost of power plants with CO_2 capture. *Int. J. Greenh. Gas Control* **1**, 188–197 (2007).
11. Higman, C. *GSTC syngas database: 2017 update*. Gasification & Syngas Technologies Conference (2017).
12. Congressional Research Service. *H.R.5376 - Inflation Reduction Act of 2022*. https://www.congress.gov/bill/117th-congress/house-bill/5376/text (2022).
13. Hydrogen Councial and McKinsey & Company. *Hydrogen insights 2023*. https://hydrogencouncil.com/wp-content/uploads/2023/05/Hydrogen-Insights-2023.pdf (2023).

14. Previsic, M. *Economic Methodology for the Evaluation of Emerging Renewable Technologies*. RE Vision Consulting, LLC: Sacramento, CA, USA (2011).
15. National Energy Technology Laboratory. *Quality Guidelines for Energy System Studies: Fuel Prices for Selected Feedstocks in NETL Studies*. Report No. NETL-PUB-22458 (2019).
16. Internal Revenue Service. *Publication 946 How to Depreciate Property*. Catalog No. 13081F (2016).
